# The mouse and ferret models for studying the novel avian-origin human influenza A (H7N9) virus

**DOI:** 10.1186/1743-422X-10-253

**Published:** 2013-08-08

**Authors:** Lili Xu, Linlin Bao, Wei Deng, Hua Zhu, Ting Chen, Qi Lv, Fengdi Li, Jing Yuan, Zhiguang Xiang, Kai Gao, Yanfeng Xu, Lan Huang, Yanhong Li, Jiangning Liu, Yanfeng Yao, Pin Yu, Weidong Yong, Qiang Wei, Lianfeng Zhang, Chuan Qin

**Affiliations:** Institute of Laboratory Animal Sciences, Chinese Academy of Medical Sciences (CAMS) & Comptive Medicine Center, Peking Union Medical Collage (PUMC), Key Laboratory of Human Disease Comptive Medicine, Ministry of Health, Beijing, China

**Keywords:** H7N9, Animal model, BALB/c mouse, Ferret

## Abstract

**Background:**

The current study was conducted to establish animal models (including mouse and ferret) for the novel avian-origin H7N9 influenza virus.

**Findings:**

A/Anhui/1/2013 (H7N9) virus was administered by intranasal instillation to groups of mice and ferrets, and animals developed typical clinical signs including body weight loss (mice and ferrets), ruffled fur (mice), sneezing (ferrets), and death (mice). Peak virus shedding from respiratory tract was observed on 2 days post inoculation (d.p.i.) for mice and 3–5 d.p.i. for ferrets. Virus could also be detected in brain, liver, spleen, kidney, and intestine from inoculated mice, and in heart, liver, and olfactory bulb from inoculated ferrets. The inoculation of H7N9 could elicit seroconversion titers up to 1280 in ferrets and 160 in mice. Leukopenia, significantly reduced lymphocytes but increased neutrophils were also observed in mouse and ferret models.

**Conclusions:**

The mouse and ferret model enables detailed studies of the pathogenesis of this illness and lay the foundation for drug or vaccine evaluation.

**Electronic supplementary material:**

The online version of this article (doi:10.1186/1743-422X-10-253) contains supplementary material, which is available to authorized users.

In March 2013, a novel avian-origin H7N9 subtype influenza virus was recognized as the causative agent of influenza-like illnesses in humans in Eastern China [1]. Most patients presented with respiratory infections that progressed to severe pneumonia and dyspnea [1]. By August 6, there are 132 human infections, with 43 fatal, identified in 10 provinces on mainland of China, and one imported case was also found in Taiwan. It is an urgency to establish animal models for this virus to evaluate drug or vaccines, and lay the foundation for further pathogenic mechanism research. In this study, BALB/c mouse and ferret (*Mustela putorius furo*) models were chosen for animal model establishment for H7N9 virus.

Mice do not run fevers or develop easy-to-measure respiratory clinical signs post inoculated with influenza viruses, however, mice display numerous physical signs of illness (including mortality, weight loss, ruffled fur, and lethargy) that can be used as indicator of pathogenicity. Female 6-week-old specific pathogen-free BALB/c mice used in this study were obtained from the Institute of Laboratory Animal Sciences, Beijing, China. Mice were anesthetized and inoculated intranasally with 50 μl of A/Anhui/1/2013 (H7N9) virus. Three groups of ten mice were septely inoculated with 10^8^, 10^7^, or 10^6^ 50% tissue culture infectious dose (TCID_50_) of H7N9 virus, and were observed daily for signs of disease and mortality up to 14 days. The percentages of mice that survived during the observation period were shown in Figure 1A. All of the mice inoculated with 10^6^ TCID_50_ of virus survived. In contrast, only 80% and 40% of the mice inoculated with 10^7^ or 10^8^ TCID_50_ of virus survived on 14 d.p.i. For mice inoculated with 10^6^ TCID_50_ of virus, the loss of body weight was observed from 2 d.p.i. and the peak loss reached 28.9% on 9 d.p.i., after which the mice began to steadily regain their body weight over the remaining observation period (Figure 1B and Table 1). Meanwhile, ruffled fur appeared from 3 d.p.i. and the occurrence rate reached 100% from 4 d.p.i. until 14 d.p.i.(Figure 2A (i) and Table 1). Furthermore, thirty mice were also inoculated with 10^6^ TCID_50_ of H7N9 virus, and six were selected randomly and euthanized on 1, 2, 3, 5, 7 d.p.i. respectively for virus dissemination and pathology analysis. From 3 to 7 d.p.i., gross examination of the lungs revealed focal to multifocal consolidation in all inoculated mice (Figure 2A (ii)). However, gross examination of the heart, liver, spleen, kidney and brain did not reveal lesions in inoculated mice.

**Figure 1:**
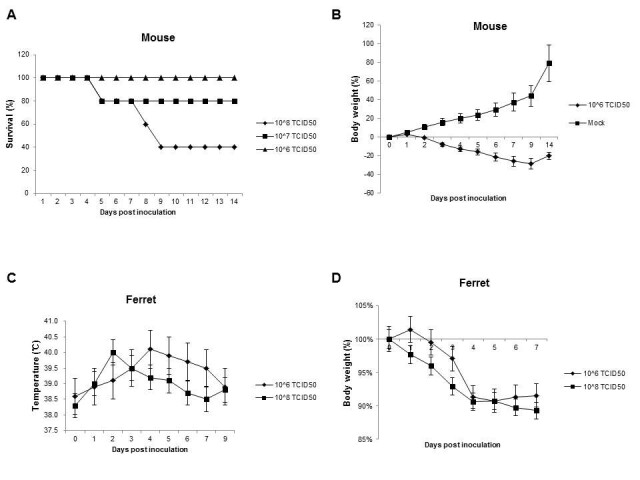
**Survival percentages and weight loss changes of inoculated BALB/c mice or ferrets with H7N9 virus. (A)** Three groups of ten mice were septely inoculated with 10^8^, 10^7^, or 10^6^ TCID_50_ of H7N9 virus, and the percentages of mice that survived during the observation period were compared. **(B)** The body weight loss of mice inoculated with 10^6^ TCID_50_ of virus. **(C)** Body temperature change of ferrets which were inoculated with either 10^8^ or 10^6^ TCID_50_ of virus. **(D)** Body weight loss of ferrets which were inoculated with either 10^8^ or 10^6^ TCID_50_ of virus. Data are presented as mean value ± SD.

**Table 1:** **Clinical signs and replication of H7N9 virus in mice and ferrets**

Mouse	Time (d.p.i.)	Clinical signs				Mean virus titer (Log_10_TCID_50_/g of tissue)							
		Weight loss (%)^*a*^	Ruffled fur^*b*^	Mortality		BALF^*c*^	Lung	Brain	Heart	Liver	Spleen	Kidney	Intestine
	1	3/10	0/10	0/10 (0%)		3.75 (4/6)^*d*^	4.15 (4/6)	-	-	-	-	-	-
	2	8/10	0/10	0/10 (0%)		2.31 (5/6)	5.69 (5/6)	-	-	-	1.25 (2/6)	1.88 (4/6)	-
	3	9/10	7/10	0/10 (0%)		2.75 (6/6)	4.38 (6/6)	-	-	-	-	-	0.69 (5/6)
	5	8/10	10/10	0/10 (0%)		3.58 (6/6)	5.38 (6/6)	-	-	0.58 (2/6)	-	-	-
	7	8/10	10/10	0/10 (0%)		2.31 (6/6)	3.69 (6/6)	-	-	-	-	-	-
**Ferret**	**Clinical signs**			**Euthanized time (d.p.i.)**	**Virus titer (Log**_**10**_ **TCID**_**50**_ **/ tissue)**								
	Weight Loss (%)	Sneezing^*b*^	Lethality^*c*^		Trachea	Lung	Brain	Heart	Liver	Spleen	Kidney	Intestine	Olfactory bulb
	9/9 (10.7/9.3)^*e*^	9/9	0/9	3	4.92 (2/2)^*d*^	2.59 (2/2)	-	2.82 (1/2)	-	-	-	-	5.41 (2/2)
				7	4.11 (2/2)	2.87 (2/2)	-	-	2.16 (1/2)	-	-	-	-

^*a*^ The percentage mean maximum weight loss after inoculation.

^*b*^ Number of animals in which ruffled fur (mouse) or sneezing (ferret) was observed after inoculation.

^*c*^ Virus titer was calculated as Log_10_ TCID_50_/ml of BALF.

^*d*^ –Numbers of positive animals in total animals.

^*e*^ 10.7% for animals inoculated with 10^8^ TCID_50_ of H7N9 virus, and 9.3% for animals inoculated with 10^6^ TCID_50_ of H7N9 virus.

Mice were anesthetized and inoculated intranasally with 50 μl of 10^6^ TCID_50_ of A/Anhui/1/2013 (H7N9) virus and were observed daily for signs of disease and mortality up to 14 days. Meanwhile, six mice were selected randomly and euthanized on 1, 2, 3, 5, and 7 d.p.i. respectively for virus dissemination analysis. Ferrets were divided into two groups, and three were inoculated with 10^8^ while other six were inoculated with 10^6^ TCID_50_ of A/Anhui/1/2013 (H7N9) virus. Two randomly selected animals which were inoculated with 10^6^ TCID_50_ of virus were euthanized septely on 3 and 7 d.p.i., and used for virus replication in ferret tissues. All nine animals were observed for clinical signs and weighed daily as an indicator of disease. Nasal and throat swabs were collected on 1, 3, 5, 7, and 9 d.p.i. and virus titers were determined by end-point titration in MDCK cells. All data are presented as mean value ± SD.

**Figure 2:**
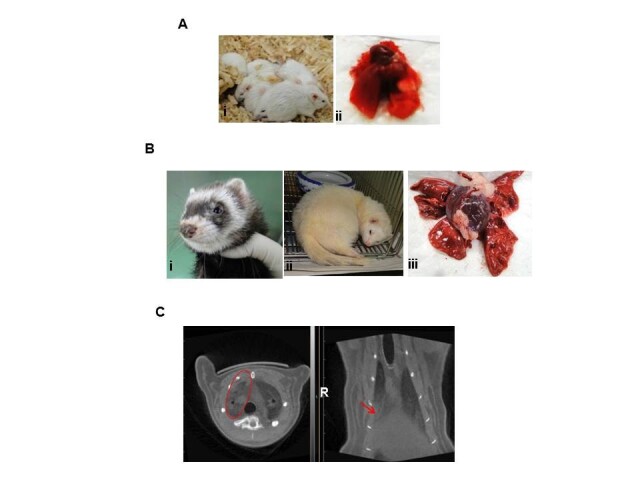
**Clinical signs and gross examination of lung tissues of inoculated BALB/c mice or ferrets with H7N9 virus. (A)** Clinical signs **(i)** and gross examination of the lungs **(ii)** of inoculated mice. **(B)** Clinical signs **(i** &**ii)**, gross examination of the lungs **(iii)** of inoculated ferrets. **(C)** Micro-CT scanning of lungs of inoculated ferrets, the lesions were marked by ovals and arrows.

The virus dissemination in the bronchoalveolar lavage fluid (BALF), lung, and other main tissues (heart, liver, spleen, kidney, intestine, and brain) of inoculated mice were titrated on MDCK cell. Virus shedding was observed to start on 1 d.p.i. and continued until 7 d.p.i. in both BALF and lungs, with lung tissues containing higher virus titers than BALF from 2 d.p.i. (*P*<0.05). The peak virus shedding reached 10^3.75^ TCID_50_ for BALF on 1 d.p.i. and 10^5.69^ TCID_50_ for lung on 2 d.p.i. (Figure 3A and Table 1). Meanwhile, virus could also be isolated from the brain, liver, spleen, kidney and intestine (Table 1). The brain tropism of H7N9 virus was coincidence with other highly pathogenic avian viruses such as H5N1and H7N7 [2–4].

**Figure 3:**
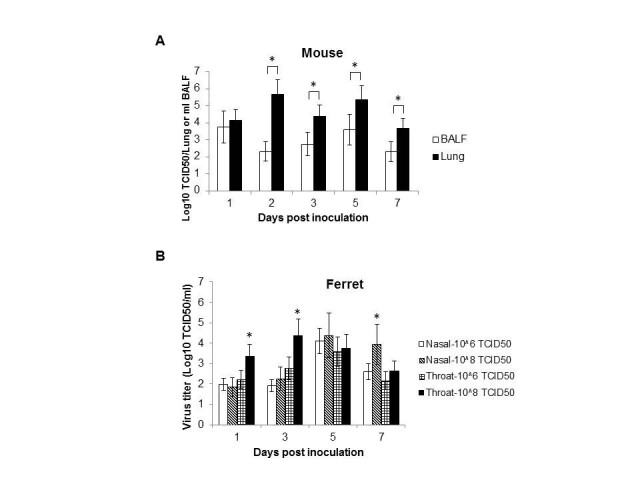
**Virus shedding of inoculated BALB/c mice or ferrets with H7N9 virus. (A)** Virus shedding in the BALF and lung tissues of inoculated mice. **(B)** Virus shedding in the nasal and throat swabs of inoculated ferrets. Data are presented as mean value ± SD. * *P*<0.05.

The dissemination of virus in inoculated mice were also assessed by immunohistochemical analysis (IHC), the viral antigens were mainly located within the epithelial cells of the bronchial in the lung, the choroid plexus in the brain, the villous column in the small intestine, and the renal tubules in the kidney (Figure 4A (i-iv)). Meanwhile, all tissues of inoculated mice were subjected to pathological analysis. Characterization of inflammation in the lungs revealed that from 1 d.p.i., lung tissues exhibited characteristic pathology of influenza infection, including inflammatory hyperaemia and exudative pathological changes. From 3 to 7 d.p.i., the lesions of lung tissue became larger, and more severe interstitial pneumonia were observed (Figure 4A (v-viii)).

**Figure 4:**
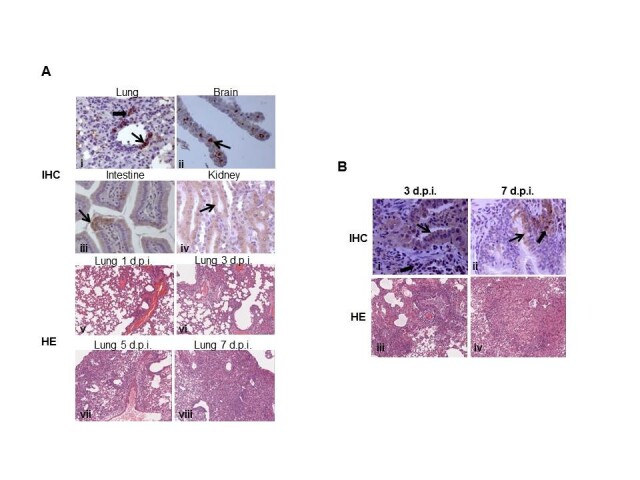
**Histopathological analyses of tissues of inoculated mice and ferrets. (A)** Hematoxylin and Eosin **(H**-**E)** stain and immunohistochemical (**IHC**) analyses of tissues of inoculated mice. **(B)** Hematoxylin and Eosin **(H**-**E)** stain and **IHC** analyses of lungs of inoculated ferrets.

On 14 d.p.i., sera from inoculated mice were collected and tested for H7N9 virus specific antibodies by using HI assay. All inoculated mice showed seroconversion, and the HI titers ranged from 80 to 160.

It has been reported that infection with H7N9 influenza viruses can cause leukopenia in clinical patients [1, 5, 6]. To determine the extent to which infection with H7N9 virus induced leukopenia in mice, peripheral blood leukocytes in inoculated mice were counted. In comparison with those in the mice before inoculation, the total numbers of white blood cells (WBCs) in the H7N9 virus inoculated mice were reduced significantly (*P*<0.05) from 2 to 7 d.p.i. Significantly reduced numbers of lymphocytes but increased numbers of neutrophils (*P*<0.05) were also observed on 2 d.p.i. in the inoculated mice (Table 2).

**Table 2:** **Impact of viral infection on the mouse and ferret lymphocyte populations in whole blood**

Animal	Time (d.p.i.)	WBC^***a***^	% LY^***b***^	% NE^***b***^
Mouse	-1^*c*^	12.0	78.4	12.5
	1	11.4	64.4	23.3
	2	6.9*	48.7*	43.8*
	3	5.5*	62.5	27.1
	5	6.8*	77.1	15.7
	7	5.3*	64.6	21.5
Ferret	-1	15.9	64.8	17.9
	7	8.4*	38.8*	57.6*
	14	13.4	55.1	37.7*

^*a*^ Number of white blood cells (WBC) in whole blood, expresses as thousands of WBCs per microliter of whole blood.

^*b*^ Mean percentage of leukocytes that are lymphocytes (LY) or neutrophils (NE) from 6 mice per group.

^*c*^ -1 d.p.i.: the day before inoculation.

* Statistical significance: * *P*<0.05 compared to the values of -1 d.p.i..

Ferret (*Mustela putorius furo*) model was also established for H7N9 virus studies. Ferrets have been used in influenza research since 1933 because they are susceptible to infection with human and avian influenza viruses [7]. After inoculation with human influenza A virus, ferrets develop respiratory disease and lung pathology similar to that observed in humans [8]. In this study, nine specific pathogen-free castrated adult ferrets, 6 to 12 months of age that were serologically negative by HI assay for currently circulating influenza viruses, were randomly divided into two groups. One group included three ferrets, which were inoculated intranasally with 10^8^ TCID_50_ of A/Anhui/1/2013 (H7N9) virus, were used for taking chest radiographs daily. Another group included left six ferrets, which were inoculated intranasally with 10^6^ TCID_50_ of A/Anhui/1/2013 (H7N9) virus. Two randomly selected animals were euthanized septely on 3 and 7 d.p.i., and used for pathological and virological examination of the trachea, lung, brain, heart, liver, spleen, kidney, stomach, intestine, and olfactory bulb. All nine animals were observed for clinical signs and weighed daily as an indicator of disease. Nasal and throat swabs were collected on 1, 3, 5, 7, 9 d.p.i. and transferred to 1 ml of phosphate buffer solution (PBS). Virus titers were determined by end-point titration in MDCK cells.

Results showed that both doses caused fever (Figure 1C), weight loss (Figure 1D), sneezing (Figure 2B (i)), lethargy (Figure 2B (ii)), decreased appetite for food in ferrets. For ferret inoculated with 10^8^ TCID_50_ of virus, the mean highest body temperature and maximum weight loss was 40.0°C on 2 d.p.i. and 10.7% on 7 d.p.i., while for animals inoculated with 10^6^ TCID_50_ of virus, the corresponding data were 40.1°C on 4 d.p.i. and 9.3% on 5 d.p.i. throughout the course of 14 days (Figure 1C&D). Gross examination of the lungs revealed multifocal consolidation in all ferrets which were inoculated with 10^6^ TCID_50_ of H7N9 virus and euthanized septely on 3 and 7 d.p.i. (Figure 2B (iii)). However, gross examination of the brain, heart, liver, spleen, kidney, stomach, intestine, and olfactory bulb did not reveal lesions in inoculated ferrets.

Histopathological analyses revealed that on 3 d.p.i., lung tissue had a multifocal mild or moderate interstitial inflammatory hyperaemia and exudative pathological changes, while on 7 d.p.i., the lesions of lung tissue became larger, and fusing of multiple patchy lesions were observed (Figure 4B (iii-iv)). Immunohistochemistry was also performed to assess the presence of H7N9 influenza virus infected cells in tissues including bronchial epithelial cells and alveolar epithelial cells from infected ferrets (Figure 4B (i-ii)).

For ferrets inoculated with 10^8^ TCID_50_ of H7N9 virus, chest radiograph was taken by micro-CT scanning. Results showed that there were inflammatory lesions (mild to bilateral ground-glass opacity) in the right superior lobe from 6 d.p.i. to 14 d.p.i. (Figure 2C).

Nasal and throat swabs were collected from inoculated animals on 1, 3, 5, 7, 9 d.p.i., and virus shedding was observed to start on 1 d.p.i. for both doses, and continued until 7 d.p.i.. The peak virus shedding in ferrets inoculated with 10^8^ TCID_50_ of virus reached 10^4.38^ TCID_50_/ml on 3 d.p.i. from throat swab, while for ferrets inoculated with 10^6^ TCID_50_ of virus, the peak shedding reached 10^4.11^ TCID_50_/ml on 5 d.p.i. from nasal swab (Figure 3B).

Parts of the tissues from euthanized ferrets were homogenized and virus titers were determined. For animals euthanized on 3 d.p.i., virus could be detected in the lung (10^2.59^ TCID_50_/gram), trachea (10^4.92^ TCID_50_/gram), and olfactory bulb (10^5.41^ TCID_50_/gram) of both ferrets, and heart (10^2.82^ TCID_50_/gram) of one ferret. On 7 d.p.i., the H7N9 virus could be isolated from the trachea (10^4.11^ TCID_50_/gram) and lung (10^2.87^ TCID_50_/gram) of both ferrets, and liver (10^2.16^ TCID_50_/gram) of one ferret (Table 1).

On 14 d.p.i. sera from inoculated ferrets were collected and tested for H7N9 virus specific antibodies by using HI assay. All inoculated ferrets showed seroconversion, and the HI titers ranged from 160 to 1280.

Meanwhile, leukopenia, significantly reduced numbers of lymphocytes but increased numbers of neutrophils (*P*<0.05) were also observed on 7 d.p.i. in the inoculated ferrets (Table 2).

Collectively, H7N9 virus caused typical clinical symptoms in both BALB/c mice and ferrets, and virus shedding or replication could also be detected in respiratory tract and other tissues, which was generally coincidence with recent other reports [9–12]. However, Mok et al. did not detected H7N9 virus dissemination beyond the respiratory tract of mice which were inoculated with 10^5^ PFU of A/Shanghai/2/2013 virus [12]. The discrepancy of virus distribution results between us may be caused by the different choices in inoculated virus and dosage. Meanwhile, Belser et al. found that systemic spread of virus to liver and heart could not been detected in inoculated ferrets [9]. We supposed that the different species of ferrets used for studies may be the main cause of discrepant results between us. These animal models establish the causal relationship between H7N9 virus and respiratory disease in both mouse and ferret reminiscent of the respiratory disease observed in humans, thus fulfilling Koch’s postulates. The mouse and ferret models enables detailed studies of the pathogenesis of this illness and lay the foundation for drug or vaccine evaluation.

## Materials and methods

### Viruses

Influenza virus A/Anhui/1/2013 (H7N9) was isolated from the third case of laboratory-confirmed human A (H7N9) virus, with Q226L mutation in the receptor binding domain of HA protein. The patient was a 35-year-old woman who lived in Anhui Province of China. She had visited a chicken market one week before the onset of symptoms. ARDS, septic shock, and acute renal damage developed on day 6 post the onset of illness. The patient was treated with oseltamivir (started on day 8), but eventually died on 19 days after the onset of illness [1]. A throat swab was collected from the patient and propagated in the allantoic sac and amniotic cavity of 9-to-11-day old embryonated chicken eggs, then passaged once in Madin-Darby canine kidney (MDCK) cells.

### Cells

MDCK cells were maintained in Eagle’s minimal essential medium (MEM, Invitrogen) in all instances supplemented with 10% fetal bovine serum (FBS), 100 IU/ml penicillin, 100 μg/ml of streptomycin and cultured at 37°C with 5% CO_2_.

### Animal models

#### Mouse

Female 6-week-old specific pathogen-free BALB/c mice used in this study were obtained from the Institute of Laboratory Animal Sciences, Beijing, China. Mice were anesthetized and inoculated intranasally with 50 μl of A/Anhui/1/2013 (H7N9) virus. Three groups of ten mice were septely inoculated with 10^8^, 10^7^, or 10^6^ 50% tissue culture infectious dose (TCID_50_) of H7N9 virus, and were observed daily for signs of disease and mortality up to 14 days. The status of weight loss was evaluated by monitoring the rate of weight change, which is calculated as follows: [(value at day of monitoring - value at 0 days post inoculation (d.p.i.))/value at 0 d.p.i.] ×100%. Ruffled fur was recorded daily and used as the standard of clinical changes, and the morbidity rate was calculated as the number of mice exhibiting ruffled fur against the monitored animals. Meanwhile, thirty mice were also inoculated with 10^6^ TCID_50_ of H7N9 virus, and six were selected randomly and euthanized on 1, 2, 3, 5, and 7 d.p.i. respectively for virus dissemination and pathology analysis.

#### Ferret

Nine specific pathogen-free castrate adult ferret (*Mustela putorius furo*), 6 to 12 months of age that were serologically negative by haemagglutinin inhibition (HI) assay for currently circulating influenza viruses, were randomly divided into two groups. One group included three ferrets, which were inoculated intranasally with 400 μl of 10^8^ TCID_50_ of A/Anhui/1/2013 (H7N9) virus, were used for taking chest radiographs daily. Another group included left six ferrets, which were inoculated intranasally with 400 μl of 10^6^ TCID_50_ of A/Anhui/1/2013 (H7N9) virus. Two randomly selected animals were euthanized septely on 3 and 7 d.p.i., and used for pathological and virological examination of the trachea, lung, brain, heart, liver, spleen, kidney, stomach, intestine, and olfactory bulb. All nine animals were observed for clinical signs and weighed daily as an indicator of disease. Nasal and throat swabs were collected on 1, 3, 5, 7, 9 d.p.i. and transferred to 1 ml of phosphate buffer solution (PBS). Virus titers were determined by end-point titration in MDCK cells.

The experimental protocol was evaluated and approved by the Institute of Animal Use and Care Committee of the Institute of Laboratory Animal Science, Peking Union Medical College (ILAS-PC-2013-009). All experiments were performed under ABSL-3 conditions.

### Virus titrations

Virus titrations were performed by end-point titration in MDCK cells. MDCK cells were inoculated with tenfold serial dilutions of homogenized tissues, nasal and throat swabs in 96-well plates. One hour after inoculation, cells were washed once with PBS and grown in 200 μl of infection media, consisting of MEM supplemented with 100 IU/ml penicillin, 100 μg/ml streptomycin, and 1 μg/ml TPCK-trypsin. At 3 d.p.i., the supernatants of infected cell cultures were tested for agglutinating activity using turkey erythrocytes as an indicator of infection of the cells. Infectious titers were calculated from five replicates by the method of Reed and Muench [13].

### Histopathology and immunohistochemistry

Animal necropsies were performed according to a standard protocol. Samples for histological examination were stored in 10% neutral-buffered formalin (lungs after inflation with formalin), embedded in pffin, sectioned at 4 μm, and stained with hematoxylin and eosin (H&E) for examination by light microscopy or with an immunohistochemical method using a monoclonal antibody against the nucleoprotein of influenza A virus (1:200 dilution, IRR Ltd, Catalogue No: FR-51) at 4°C overnight. The sections were washed three times with PBS and then incubated with HRP-conjugated goat anti-mouse secondary antibody (1:5000 dilution, Sigma, Catalogue No: PV-9002). The sections were developed with 3-3’ diaminobenzidine (DAB) and examined with a light microscope.

### Hematology analysis

The total white blood cell (WBC) counts and lymphocytes in individual heparinized blood samples were determined on an ACT TM laser-based hematology analyzer (Beckman Coulter, USA).

### Micro-CT scanning

Scans were performed using a cone-beam micro-CT scanner (Inveon, Siemens Healthcare, Germany). Inoculated ferrets were anesthetized and placed prone position on the micro-CT bed without respiratory gating. The tube voltage was 70 kVp and current was 400 mA, and exposure time was 800 ms. The scan field of view (FOV) was 72.44 mm × 71.31 mm. Projections images were acquired using a single tube/detector over a circular orbit of 360° with a step angle of 1°. Reconstructions were performed using a commercially available CT reconstruction program (COBRA Exxim, v6.3), with a filtered back projection technique. A resolution of approximately 70.74 μ/pixel was achieved.

### HI assays

Standard HI assays were performed on post-exposure ferret sera using 0.5% turkey erythrocytes in accordance with WHO guidelines with established procedures [14]. Sera were collected from inoculated animals and tested for H7N9 virus specific antibodies.

### Statistical analysis

The difference in ferret body weight and virus titers among different groups was analyzed by one-way ANOVA and post hoc. Bonferroni correction analysis and the difference between two groups were analyzed by Student’s *t*-test using SPSS 11.5 software. A probability value of <0.05 was considered as statistically significant.

## Authors’ original submitted files for images

Below are the links to the authors’ original submitted files for images. Authors’ original file for figure 1 Authors’ original file for figure 2 Authors’ original file for figure 3 Authors’ original file for figure 4
