# Correction to: The mouse and ferret models for studying the novel avian-origin human influenza A (H7N9) virus

**DOI:** 10.1186/s12985-020-01356-4

**Published:** 2020-06-24

**Authors:** Lili Xu, Linlin Bao, Wei Deng, Hua Zhu, Ting Chen, Qi Lv, Fengdi Li, Jing Yuan, Zhiguang Xiang, Kai Gao, Yanfeng Xu, Lan Huang, Yanhong Li, Jiangning Liu, Yanfeng Yao, Pin Yu, Weidong Yong, Qiang Wei, Lianfeng Zhang, Chuan Qin

**Affiliations:** Institute of Laboratory Animal Sciences, Chinese Academy of Medical, Sciences (CAMS) & Comparative Medicine Center, Peking Union Medical, Collage (PUMC), Key Laboratory of Human Disease Comparative Medicine, Ministry of Health, Beijing, China

**Correction to: Virol J 10, 253 (2013)**

**https://doi.org/10.1186/1743-422X-10-253**

Following publication of the original article [1], the authors identified an error in Fig. 4. The immunohistochemical result of intestine of inoculated mouse was unfortunately misplaced, although the error would not change the interpretation and conclusions of this work. The wrong photo has been corrected in the new figure. Scale bars of each image were also complemented.

The correct figure is given below.

**Fig. 4:**
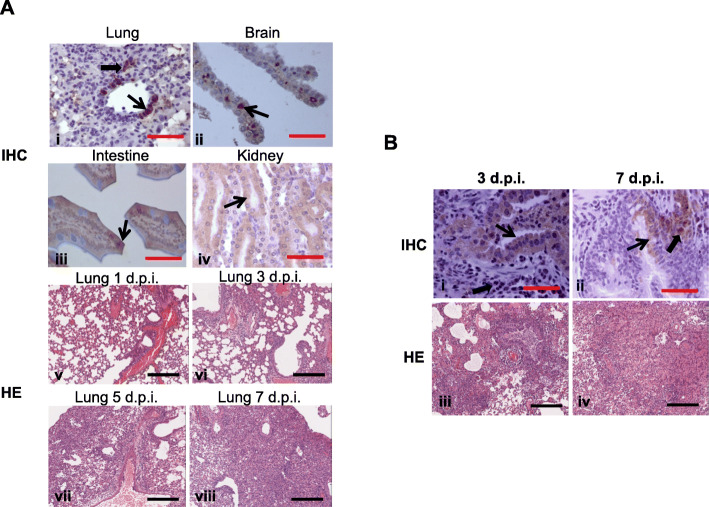
Histopathological analyses of tissues of inoculated mice and ferrets. (A) Hematoxylin and Eosin (H-E) stain and immunohistochemical (IHC) analyses of tissues of inoculated mice. (B) H-E stain and IHC analyses of lungs of inoculated ferrets. Red scale bar for IHC = 50 μm. Black scale bar for H-E = 200 μm
