# Supplementary figures and images for: The mouse and ferret models for studying the novel avian-origin human influenza A (H7N9) virus
Source: Virol J. 2013 Aug 8;10:253. doi: 10.1186/1743-422X-10-253 (PMC3750532; doi:10.1186/1743-422X-10-253)

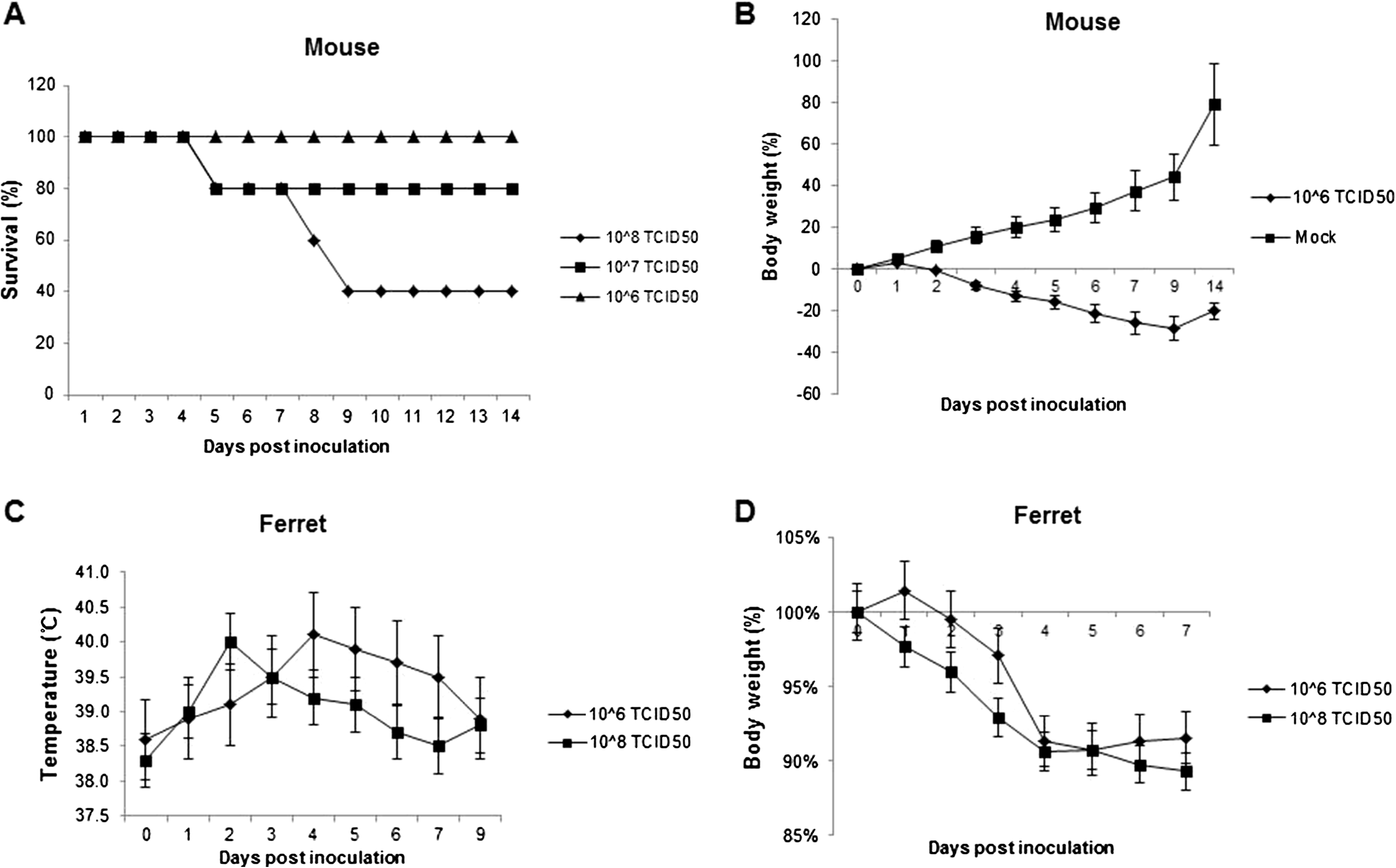

Supplement: Supplementary file 1 — Authors’ original file for figure 1 [file 12985_2013_2239_MOESM1_ESM.tif]

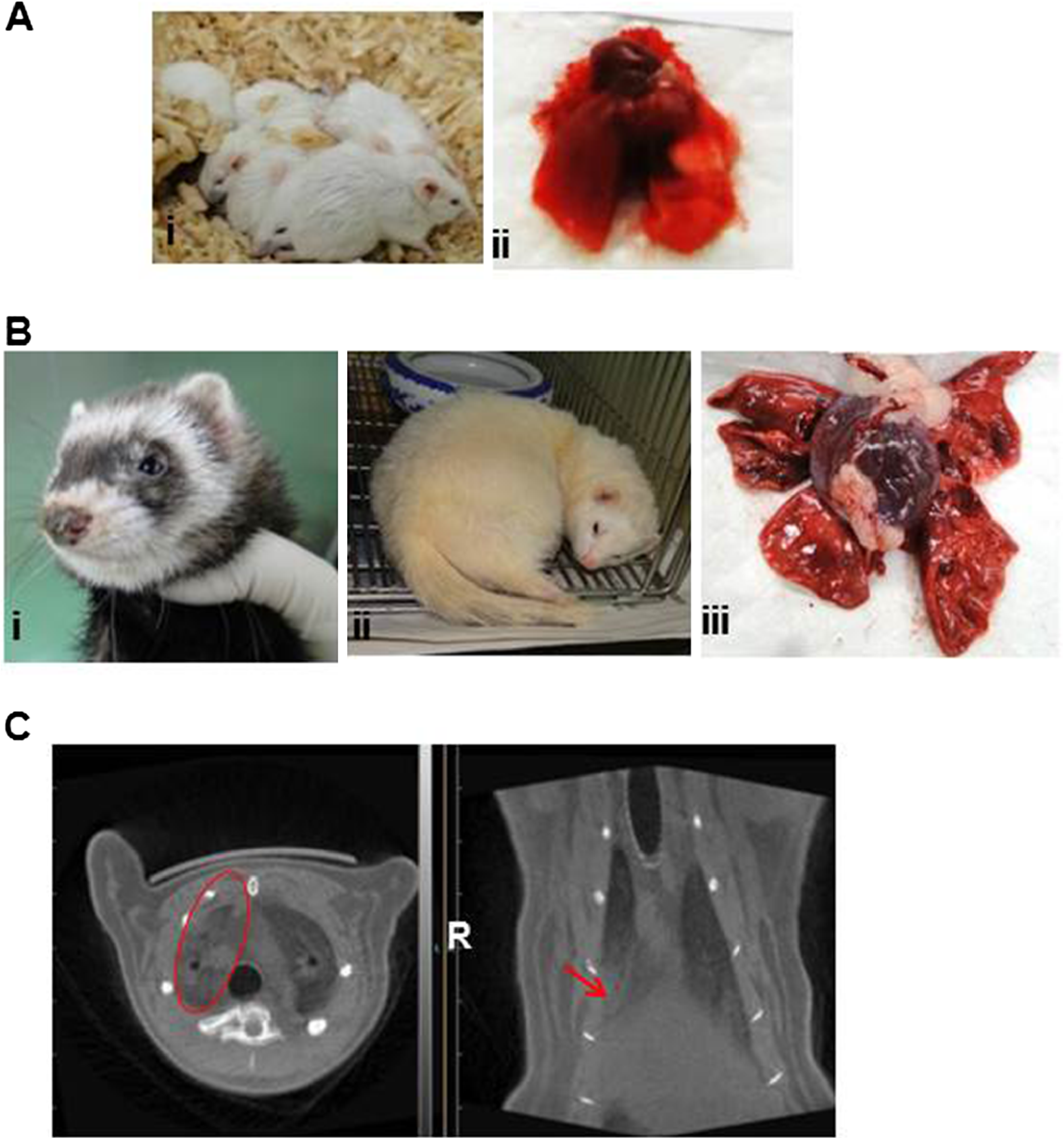

Supplement: Supplementary file 2 — Authors’ original file for figure 2 [file 12985_2013_2239_MOESM2_ESM.tif]

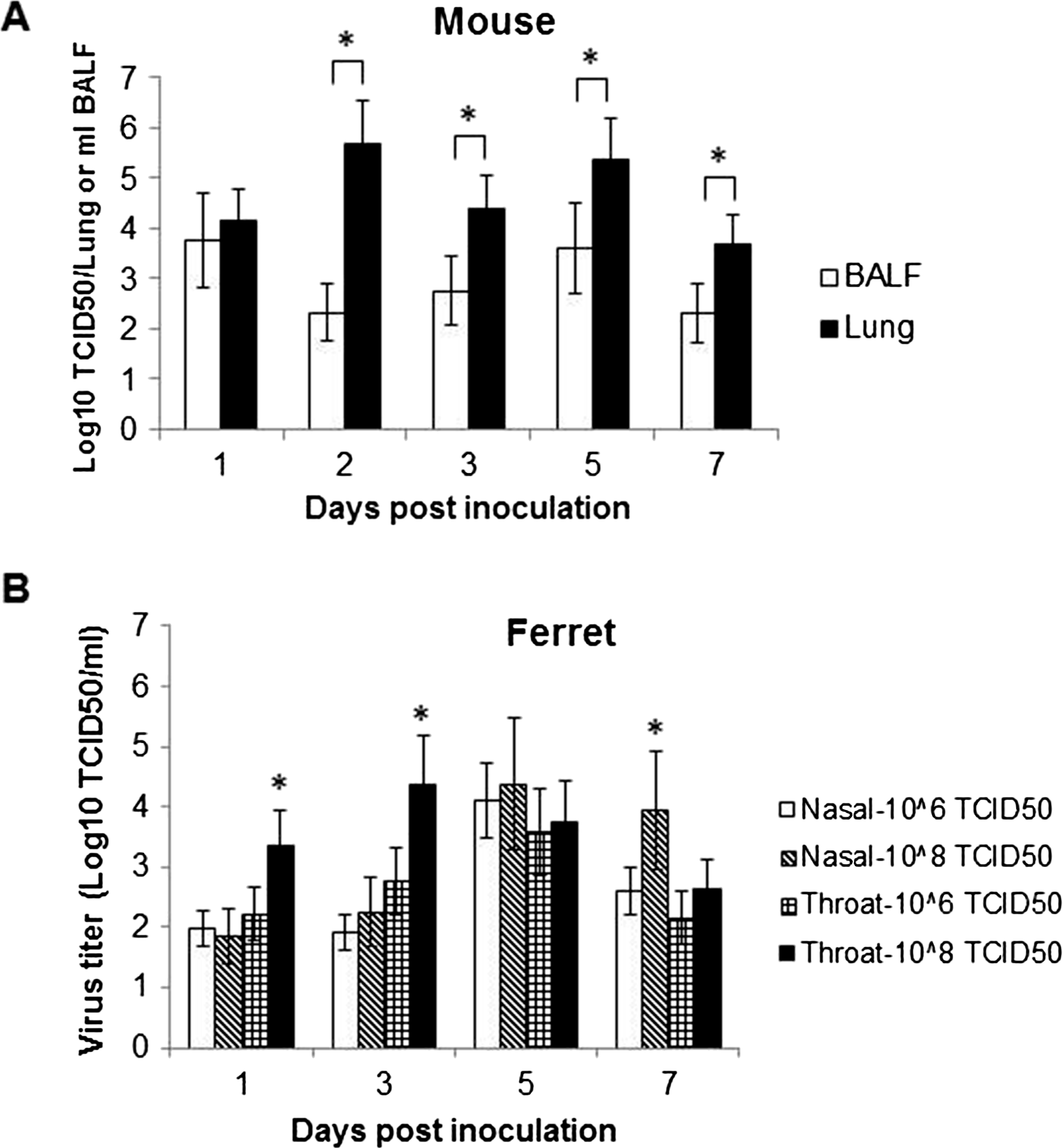

Supplement: Supplementary file 3 — Authors’ original file for figure 3 [file 12985_2013_2239_MOESM3_ESM.tif]

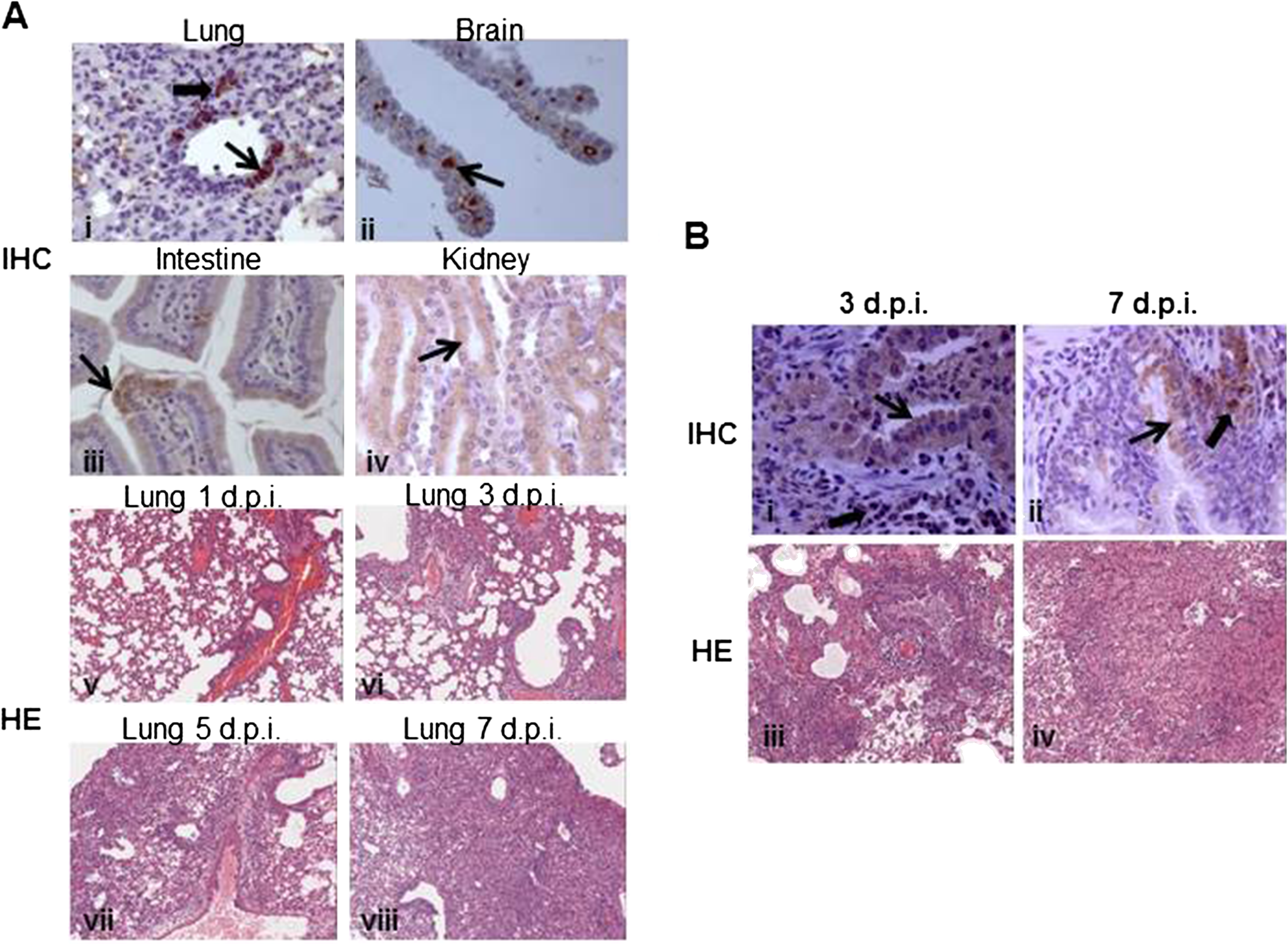

Supplement: Supplementary file 4 — Authors’ original file for figure 4 [file 12985_2013_2239_MOESM4_ESM.tif]
